# Tick-borne relapsing fever Borreliosis, a major public health problem overlooked in Senegal

**DOI:** 10.1371/journal.pntd.0009184

**Published:** 2021-04-22

**Authors:** El Hadji Ibrahima Ndiaye, Fatou Samba Diouf, Mady Ndiaye, Hubert Bassene, Didier Raoult, Cheikh Sokhna, Philippe Parola, Georges Diatta

**Affiliations:** 1 Aix Marseille Univ, IRD, APHM, SSA, VITROME, Marseille, France; 2 IHU Méditerranée Infection, Marseille, France; 3 VITROME, Campus International IRD-UCAD Hann, Dakar, Senegal; 4 Laboratoire d’Entomologie, Bactériologie, Rickettsiologie, Virologie, Département de Biologie Animale, Faculté des Sciences et Techniques, Université Cheikh Anta Diop de Dakar, Dakar, Senegal; 5 Aix Marseille Univ, IRD, APHM, MEPHI, Marseille, France; Yale University, UNITED STATES

## Abstract

**Background:**

Tick-borne relapsing fever (TBRF) is the most common vector-borne bacterial disease in humans in West Africa. It is frequently clinically confused with malaria. Our study aims to determine, on a micro-geographic scale, the conditions for the maintenance and spread of TBRF in the Niakhar district of Senegal.

**Methodology/Principal findings:**

We conducted clinical, entomological and animal reservoir investigations. Field surveys were carried out in order to investigate the presence of *Ornithodoros sonrai* vector ticks and to detect *Borrelia* spp. by qPCR using the 16S rRNA and *glpQ* genes, respectively. Micromammal trapping series were carried out inside homes and *Borrelia* infection was detected using brain tissue qPCR. Capillary blood samples from febrile patients were also tested for *Borrelia* using qPCR. More than 97% (40/41) of the villages surveyed were infested with *O*. *sonrai* ticks. The prevalence of *Borrelia* spp. infections in ticks was 13% (116/910), and over 73% (85/116) were positively confirmed as being *Borrelia crocidurae*. Borreliosis cases accounted for 12% (94/800) of episodes of fever and all age groups were infected, with children and young people between the ages of 8–14 and 22–28 being the most infected by the disease (16% and 18.4%). TBRF cases occurred in all seasons, with a peak in August. In two species of small rodents that were found to be infected (*Arvicanthis niloticus*, *Mus musculus*), the proportion of *Borrelia* infection was 17.5% (10/57), and the highest prevalence of infection (40.9%, 9/22) was observed in *A*. *niloticus*.

**Conclusion/Significance:**

Our study indicates that TBRF is an endemic disease in the Niakhar district, where children and young people are the most infected. *Arvicanthis niloticus* and *O*. *sonrai* ticks are massively present and appear to be the main epidemiological reservoirs causing its extensive spread to humans.

## Author summary

Tick-borne relapsing fever (TBRF), remains an overlooked and neglected disease in the management of febrile patients in Senegal. The results of this study showed a high prevalence of rodent burrows infested with *O*. *sonrai* ticks (the vectors of the disease) inside human dwellings, with a common *Borrelia* infection affecting populations within the community in the Niakhar district of the Fatick region of Senegal. *Borrelia* infection was detected in several rodents belonging to two species of small mammals. In febrile patients studied in the area, all age groups were infected. TBRF cases were observed in all seasons. In the similar context to that of malaria pre-elimination observed in Dielmo and Ndiop villages, TBRF appears to be the predominant cause of morbidity among febrile patients. By extension, we believe this may be the same in many other endemic regions of the country which have not yet been clinically investigated. TBRF cases are probably undiagnosed in those endemic areas and are likely to be poorly managed in health posts and health centres.

## Introduction

West African tick-borne relapsing fever (TBRF), is widespread in Saharan, Sahelian and Sudano-Sahelian regions [1] where the annual average rainfall is between 50–250 mm, 250–500 mm and 500–750 mm respectively [2]. TBRF is caused by relapsing fever *Borrelia* spp. and remains poorly understood by clinicians [3]. In West Africa, TBRF is transmitted to humans through the bite of the endophilic tick *Ornithodoros sonrai* (formerly *Alectorobius sonrai*), which lives in rodent burrows but may occasionally bite outside the burrow when these open into human dwellings [1,4,5]. Small rodents and insectivores are the main reservoir hosts for relapsing fever *Borrelia* [1,6–8].

In Senegal, the known causative agent of TBRF is *Borrelia crocidurae* [1,9]. As with other TBRF agents, *B*. *crocidurae* causes a febrile disease with repeated episodes of fever. Untreated patients experience fevers that may last for several days and a series of febrile periods of up to 3–4 days may continue for weeks or even months [10,11]. Complications can occur at any time during the course of the disease, including the development of meningoencephalitis, hepato-nephritis, eye disorders and spontaneous abortions in pregnant women [10–13]. Mortality due to *B*. *crocidurae* TBRF is poorly known. While the lethality rate of infection caused by *B*. *duttonii*, in eastern, southern and central Africa is about 2–5% [10,14]. In Senegal, TBRF tick vectors are geographically distributed across the northern two-thirds of the country, north of the 750-mm isohyet, and the southern limit of the vector tick corresponds approximately to latitude 13°40’N [1]. The prevalence of *Borrelia* infection in small mammals can reach 30% [1,6–8].

**Fig 1:**
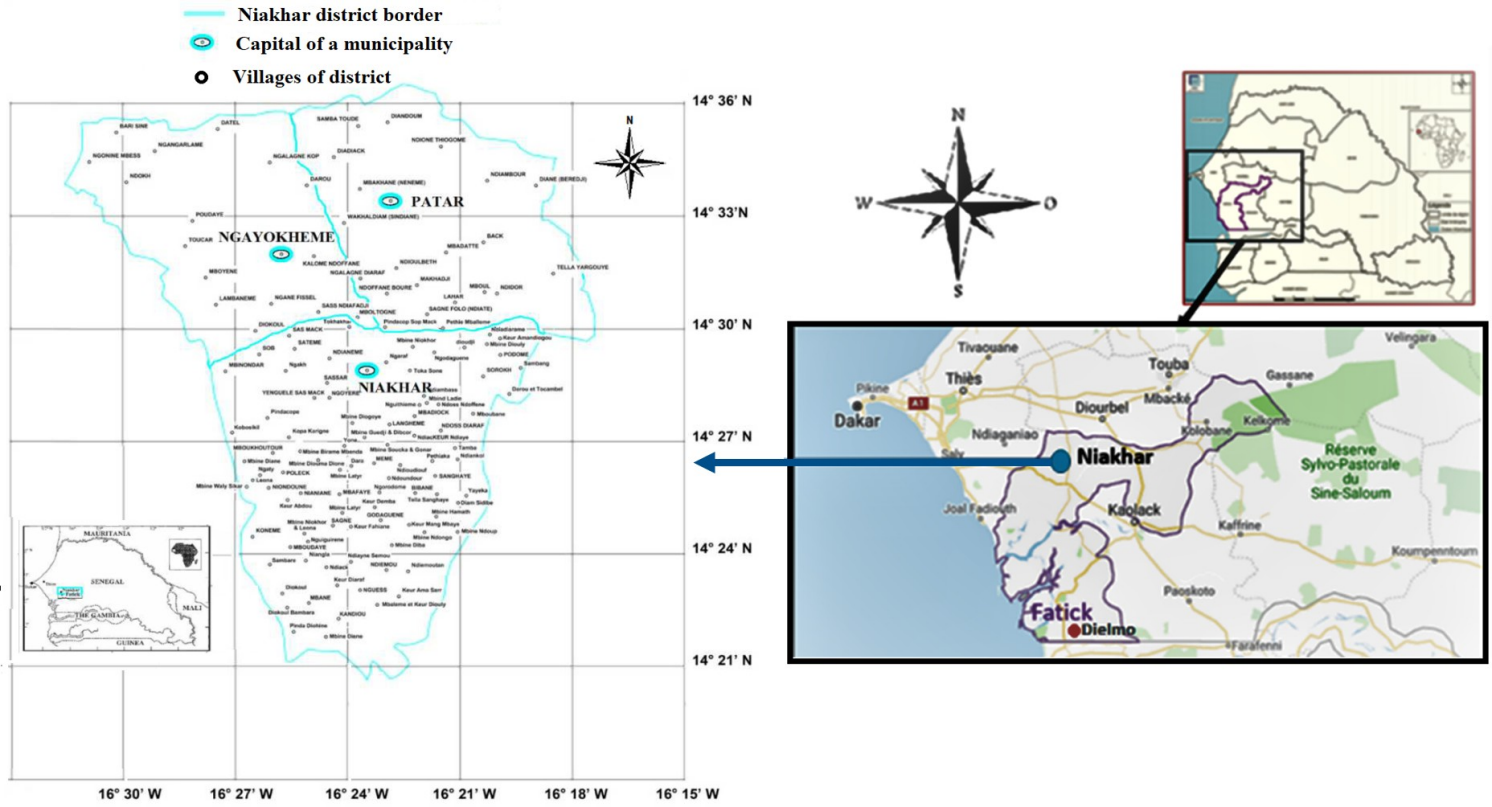
Location of the Niakhar district in the Fatick region of Senegal (source VITROME, IRD Dakar, Senegal).

Monitoring of the population of Dielmo in the Fatick region, between 1990 and 2003, (Fig 1) revealed that, on average, 11% of the population developed TBRF each year, with a prevalence of infection fluctuating between 4% and 25% depending on the year [3,5]. In this region, TBRF was the second leading cause of morbidity by vector-borne disease after malaria [3] and would currently appear to be the leading cause of morbidity in the context of malaria pre-elimination [5,15]. The diagnosis of TBRF was based on the observation of spirochetes when a thick drop blood was stained with Giemsa, and 200 oil-immersion fields (X 1,000) were systematically examined (equivalent to approximately 0.5 μl of blood) [7]. This technique, identical to that used to detect malaria haematozoa, has to be performed by a trained microscopic expert using blood taken during the febrile peak. In the free clinics in Dielmo and Ndiop, two villages in the Fatick region of Senegal, about 2% (4/206) of blood samples from febrile patients were found to be infected with *Borrelia* [16], while the same blood samples were tested in Marseille, using real-time PCR specific for the 16S rRNA *Borrelia* gene, and 13% (27/206) were found to be positive [16]. This is why, since November 2011, the thick drop blood technique which is routinely used has been coupled with a more accurate and rapid point-of-care (POC) molecular biology diagnostic laboratory, which has been set up at the Dielmo Research Station [17]. Screening for these pathogens by the POC in Dielmo between 2011 and 2016 revealed that the *B*. *crocidurae* bacterium was the main cause of consultation for febrile syndromes in the health facility, after malaria and flu [18].

Moreover, a study performed between June 2010 and October 2011 indicated a high prevalence of *Borrelia* infection in febrile patients (19.1%, 33/173) in the Niakhar area [19], where a point-of-care molecular biology laboratory was established with a design concept similar to that previously described at the research station in Dielmo [17]. In the Niakhar area, no epidemiological study has yet been performed to better understand the reasons for the frequency and occurrence of human borreliosis cases. Our study aims to determine, on the micro-geographic scale, the conditions for the maintenance and spread of TBRF in the Niakhar district and to identify the causative pathogenic species.

## Material and methods

### Ethics statement

The Senegal National Ethics Committee for Health Research (CNERS) approved this study in the context of epidemiological surveillance of fever cases (statement number # 0087).

Small mammals were treated in a humane manner, and in accordance with guidelines of the American Society of Mammalogists (Animal Care and Use Committee 2011). Sampling of rodents and insectivores excluded national parks and protected areas, did not include endangered or protected species (CITES, UICN, National guidelines), and was carried out with the authorisation of national institutions and national ministries of health. After the owner of the home was informed about the aim of the study and gave permission to conduct the study, *Ornithodoros* ticks and rodents trapping were conducted inside the home. The permission received from the homeowners was verbal.

### Study site

**Fig 2:**
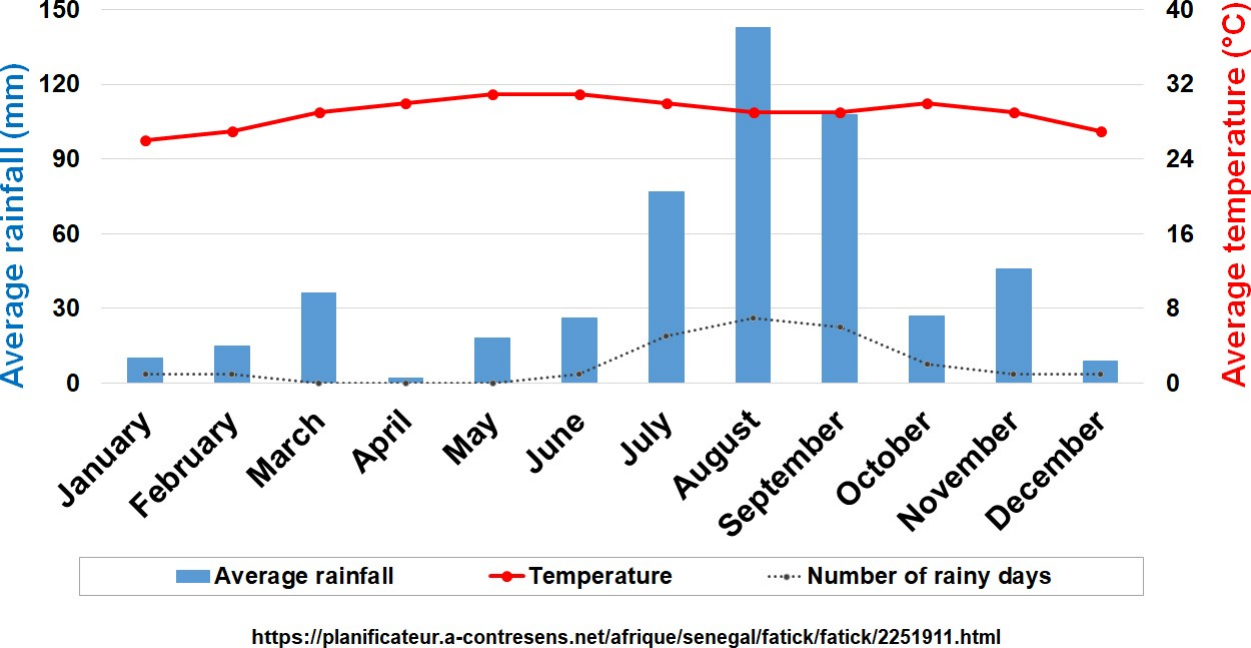
Average monthly temperature and rainfall data in the Fatick region, Senegal.

The epidemiological TBRF study was carried out in the Niakhar district, located between latitudes 14°36’N / 14°21’N and longitudes 16°18’W / 16°30’W (Fig 1), This study site falls within the Fatick region (Fig 1), 155 kilometres south-east of Dakar [18]. The area includes about 130 villages, with a population estimated at more than 55,000, of which more than half are under the age of 20 [20]. The Serer ethnic group remains the majority and represents 96.5% of the population [21]. The climate is typically Sahelian with average annual rainfall ranging between 500 mm and 600 mm between June and October added to off-season rains that were recorded during the dry season (Fig 2) [22], and temperatures are less extreme (Fig 2) compared to those in other parts of the country with the exception of the Dakar region.

### Entomological study

**Fig 3:**
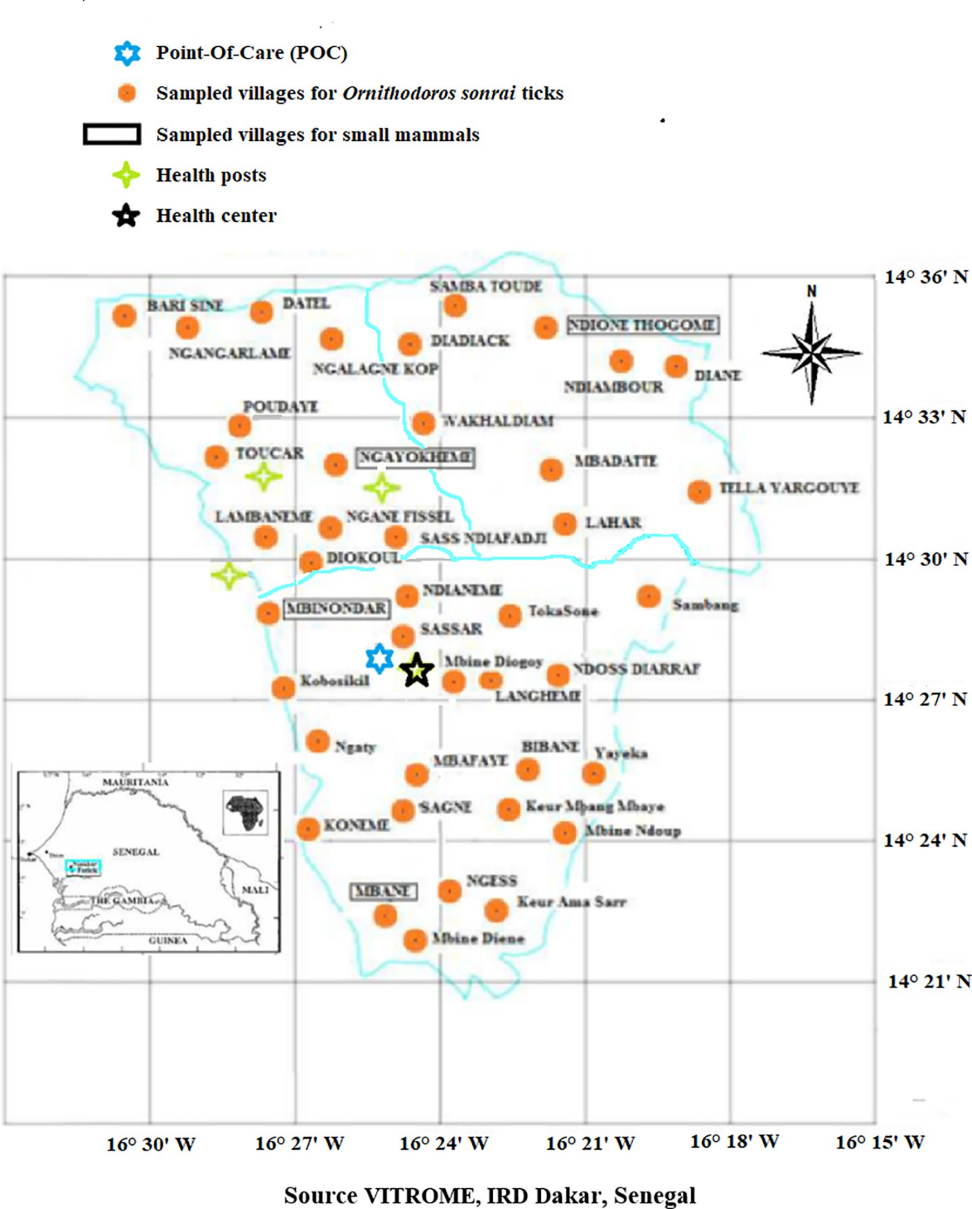
Sampling design for *O. sonrai* ticks (orange circles) and capture of small rodents and insectivores (villages in the boxes), with the presence of health posts (green star) and the health center in the Niakhar district (source VITROME, IRD Dakar, Senegal).

In August 2016, 41 villages in the Niakhar area were investigated for the presence of borreliosis *Ornithodoros* tick vectors. The choice of villages was made on the basis of the geographical distance between villages and every three minutes, ranging from latitudes 14°21′N to 14°36′N and longitudes 16°18′W to 16°30′W, a specific number of villages was chosen (Fig 3). In each village, a total of 30 rodent burrows were explored, except in some locations between infested villages which were located at various latitudes and/or longitudes where only 10 to15 positive burrows were investigated. Ticks were collected from inside rodent burrows that opened into bedrooms, kitchens, stores, and attics in human dwellings using a portable petrol-powered aspirator, as previously described [14]. All collected ticks were stored in cryotubes containing 90% ethanol and then their morphological features were examined under a binocular magnifying glass to confirm that these ticks were attributable to *O*. *sonrai* [23,24,25].

### Clinical study

Between January and December 2016, febrile patients were examined respectively in one health center and three health posts in Niakhar, Ngayokheme, Toucar, and Diohine, which are included in our Health and Demographic Surveillance System (HDSS) [22]. Each patient who came for consultation with a fever ≥ 37.5°C was systematically eligible for inclusion in this study regardless of whether the patient’s temperature was taken at the clinic. A capillary blood sample was immediately taken and sent to the POC in Niakhar to examine for pathogens, and febrile patients with a negative rapid diagnostic test for *Plasmodium* (RDT *P*.*f*) were tested for *Borrelia* infection including *Coxiella Burnetii*, *Bartonella* spp., *Leptospira* spp., *Tropheryma whipplei*, *Rickettsia* spp., malaria, dengue and influenza A and B [17]. A point-of-care clinical database containing the results of the molecular analyses made it possible to identify all causes of fever for each patient who visited these three health posts and/or the health center.

The results of the POC were transmitted to the three health posts and/or the health center for a better management of the patients using an appropriate treatment with doxycycline.

### Animal reservoir study

In October 2016, small live mammals were trapped inside human dwellings and household yards in four randomly selected villages in the Niakhar district, where *O*. *sonrai* ticks had been identified, using lattice-work traps baited with onion for 937 trap-nights (Fig 3). The traps were placed in the evening for two successive nights before being moved, each time renewing the previous day’s bait. For indoor traps, at least one or two traps were placed in each room, kitchen, attic and store. A catch report was made every morning regarding the collection of rodents. Small rodents and insectivores brought back alive to the laboratory were sacrificed using chloroform. A brain sample was taken from each animal and preserved in 90% alcohol for real-time PCR testing for *Borrelia*, as the preferential tropism of *Borrelia* for the central nervous system in reservoir hosts provides twice the number of expected positive individuals than blood samples [26].

### Molecular detection of *Borrelia* spp.

Capillary blood samples from febrile patients were subjected to DNA extraction using the QIAamp kit (QIAGEN, Hilden, Germany), as previously reported [19]. Tick samples and the brains of small mammals were subjected to DNA extraction using Cetyl Trimethyl Ammonium Bromide (CTAB) [27,28] added to proteinase K. The real-time quantitative polymerase chain reaction (qPCR) was performed using the classical *16S_Bor* rRNA system that amplifies 148 bp with primers (Bor_16S_3_F 5’ AGCCTTTAAAGCTTCGCTTGTAG 3’ and Bor_16S_3_R 5’ GCCTCCCGTAGGAGTCTGG 3’) and probe (Bor_16S_3_P 6FAM- CCGGCCTGAGAGGGTGAACGG -TAMRA) [16,29]. We consider a sample to be positive only if the sigmoid curve has a cycle threshold (Ct) lower than 36, which corresponded to the ability to reveal 10–20 copies of bacterial DNA [17,30]. Samples that tested positive for *Borrelia* spp. by *16S_Bor* rRNA were subjected to a qPCR for *glpQ* gene specific from *B*. *crocidurae* using a primer (Bcroci_glpQ_F 5’ CCTTGGATACCCCAAATCATC 3’ and Bcroci_glpQ_R 5’ GGCAATGCATCAATTCTAAAAC 3’) and probes (Bcroci_glpQ_P 6FAM ATGGACAAAATGACAGGTCTTAC-TAMRA) [31,32]. The preparation mix (Roche mix (2x) comprising nucleotide free sterile water, uracil-DNA glycosylase, primers and probe was used for negative controls, and *B*. *crocidurae* positive DNA for positive controls, as previously described [19], in the amplification analyses for each test to validate the reliability of the results.

### Statistical analysis

The analyses were performed using the R software, Version 3.3.2 (2016-10-31) and Stata, Version 11.0 [33,34]. Variation in the prevalence of indoor *O*. *sonrai* tick infestations from inside burrows and that of *Borrelia* infection was respectively compared between the sampled villages studied (when the theoretical numbers are greater than 5), between nymphs and adults, and between sex (male and female) of *O*. *sonrai* ticks tested by Pearson’s chi-squared tests. Statistical differences in the proportion between the two values was deemed significant when p < 0.05. The comparison between the age groups of febrile patients was then examined using the odds ratio test OD according to an applied logistic regression.

## Results

### Detection of *Borrelia* infection in samples of *Ornithodoros sonrai* ticks

**Fig 4:**
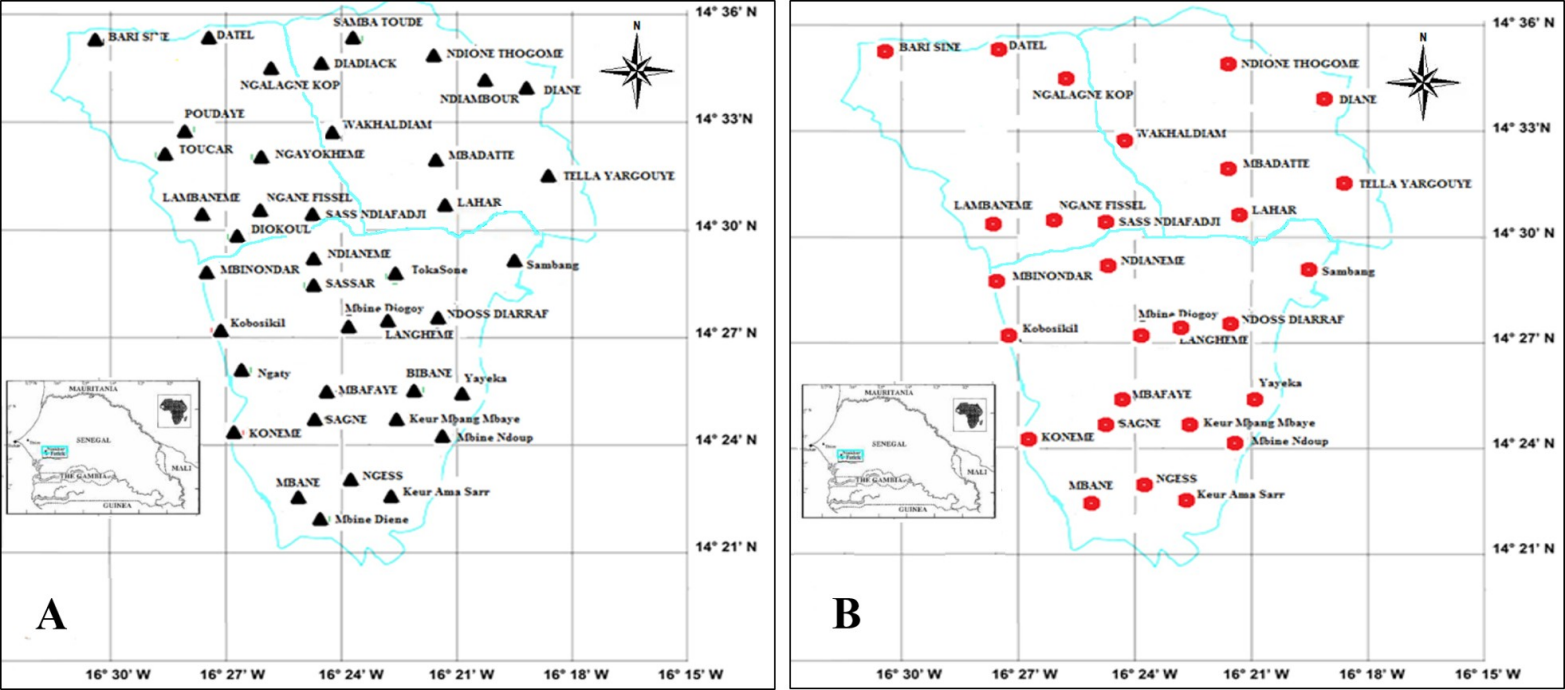
**A.** Spatial distribution of *Ornithodoros sonrai* ticks found in burrows of small mammals in the Niakhar district. **B.** Spatial distribution of *B*. *crocidurae* infection in the *O*. *sonrai* tick vector in the Niakhar district (source VITROME, IRD Dakar, Senegal).

In total, 788 rodent burrows in the human dwellings of 41 villages in the Niakhar area were examined, and more than 97% (40/41) of these villages were found to be infested with *O*. *sonrai* ticks. Of the 788 burrows surveyed, 287 were infested with *O*. *sonrai* ticks (S1 Table and Fig 4A), representing an infestation rate of 36.4% (S1 Table). The variation in the prevalence of indoor *O*. *sonrai* tick infestation of indoor burrows was significant between sampled villages, ranging from 13.2% to 66.6% (Pearson chi-squared test = 27.47; p = 0.011 < 0.05).

Of the 1,283 specimens of *O*. *sonrai* ticks collected, 910 randomly selected DNA samples from these ticks were analysed using qPCR to detect *Borrelia* spp. and *B*. *crocidurae*. At least 27.9% (80/287) of *O*. *sonrai* infested burrows presented one or more ticks infected with *Borrelia* spp. Of the 910 DNA samples tested, 116 were found to be positive for *Borrelia*, with a prevalence of 12.7%. A total of 85/116 (73.3%) were positively identified as *B*. *crocidurae* by qPCR specific glpQ for *B*. *crocidurae* (Fig 4B and Table 1). *Ornithodoros sonrai* adult ticks were more likely to be infected (Table 1) with *Borrelia* spp. (14.6%, 78/535) than nymphs (10.1%, 38/375; p < 0.05 (Pearson’s chi-squared test = 3.92; p = 0.048 < 0.05). Male *O*. *sonrai* ticks appeared to be less infected (Tables 1) with *Borrelia* spp. (12.9%, 34/263) than females (16.2%, 44/272), but the difference in *Borrelia* infection between the sexes was not statistically significant.

**Table 1:** Prevalence of *Borrelia* infection in *O*. *sonrai* ticks in the Niakhar district in Senegal.

Sex or stage	Molecular detection by qPCR		
	*16S* RNA gene *Borrelia* spp.	*glpQ* gene *B*. *crocidurae*	*P-value*
	(Number of infected ticks / Number tested)		
Males	12.9% (34/263)	79.4% (27/34)	P* < 0.05
Females	16.2% (44/272)	61.4% (27/44)	
Adults	14.6% (78/535)	69.2% (54/78)	P** > 0.05
Nymphs	10.1% (38/375)	81.6% (31/38)	
Total	12.7% (116/910)	73.3% (85/116)	

P* = *P-value between sex males and females for 16S* RNA gene *Borrelia* spp.

P** = *P-value between stage adults and nymphs for 16S* RNA gene *Borrelia* spp.

### TBRF in febrile patients of the Niakhar district

#### Prevalence of *Borrelia* infection in febrile patients

**Fig 5:**
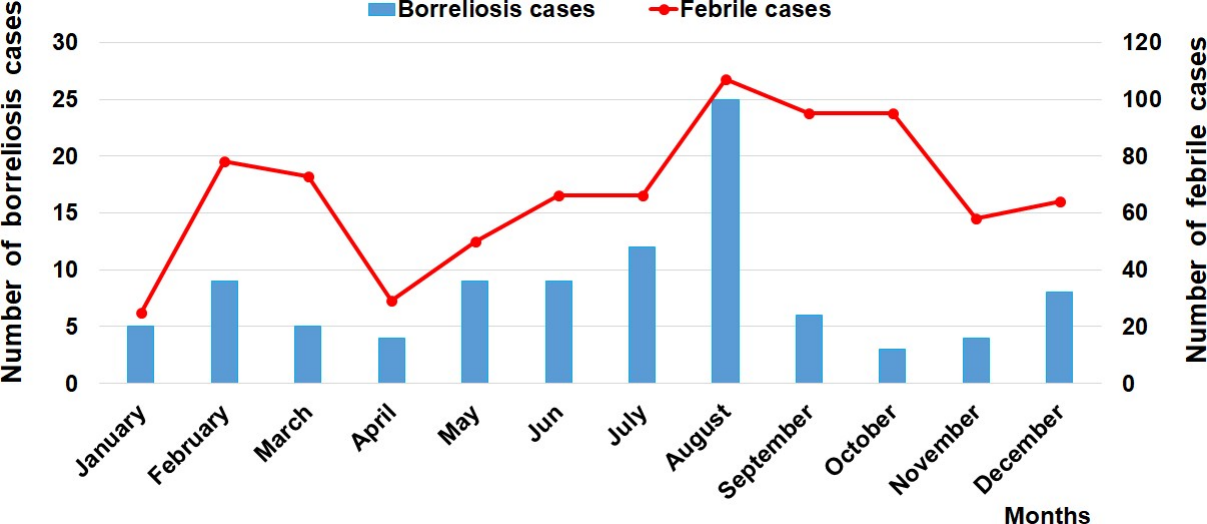
Monthly variation in visits of febrile cases and the prevalence of borreliosis cases diagnosed in the Niakhar district.

**Fig 6:**
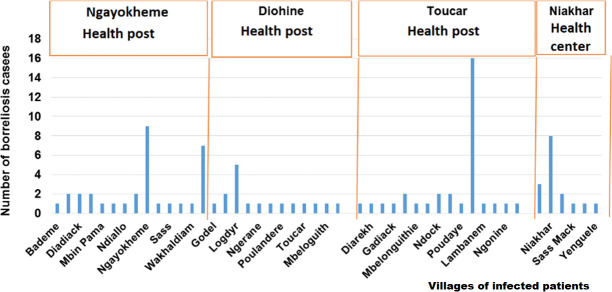
Annual distribution of diagnosed borreliosis cases by village, health post and the health center in the Niakhar district.

A total of 800 febrile patients were included in the study. A total of 94 were found to be infected by *Borrelia* spp. (11.7%). Of them, 13 patients were identified at the Niakhar health center (15.3%), 23 at the Diohine health post (7.6%), 24 in the Ngayokheme health post (13.9%), and 34 in the Toucar health post (14.9%). Patients found to be infected with *Borrelia* in these three health posts and the health center came from the surrounding villages. Cases of TBRF were observed in all seasons, with a strong seasonal trend in the distribution of cases, with most of them occurring between July and August and with a peak being observed in August (Fig 5). No mortality was reported during the study. In the three health posts and in the health center, patients mainly came from the village in which the health post and/or health center was located (Fig 6). Of the patients infected by *Borrelia* examined in the three health posts and/or the health center in the Niakhar district (Fig 6), 43 were men (45.7%, 43/94) and 51 were women (54.2%, 51/94). The average number of human cases reached during the study period was eight cases per month (standard deviation = 6), with a minimum of three and maximum of 25 cases monthly. The youngest infected febrile patients were three 1-year-old babies and the oldest was a 61-year-old adult. All age groups were infected, but the number of cases was higher among children, with 41 cases between 0–7 years and 17 cases between 8–14 years. Young adults were also infected, with 10 cases occurring between 15–21 years with a low proportion of infection, as well as seven cases between 22–28 years old and, finally, 14 cases in adults over the age of 29 and (Table 2). A significant difference in *Borrelia* infection was noted between age groups, and this was demonstrated for the 8–14 age group, with an OR rating ratio > 1 according to a logistic regression determining an infected factor.

**Table 2:** Prevalence of *Borrelia* infection by age group in the Niakhar district.

Health posts and/or Health center	Age groups				
	0–7 years	8–14 years	15–21 years	22–28 years	29 years and over
Niakhar	10.3% (6/58)	41.6% (5/12)	0% (0/6)	14.2% (1/7)	0% (0/2)
Diohine	5.5% (11/200)	9.4% (3/32)	11.1% (3/27)	9% (1/11)	12.9% (4/31)
Ngayokheme	9.8% (8/81)	12.5% (3/24)	9.5% (2/21)	27.3% (3/11)	14.3% (5/35)
Toucar	13.7% (16/116)	15.8% (6/38)	13.9% (5/36)	22.2% (2/9)	17.8% (5/28)
Total	9% (41/455)	16% (17/106)	11.1% (10/90)	18.4% (7/38)	14.6% (14/96)

### TBRF *Borrelia* infection in an animal reservoir in the Niakhar district

**Fig 7:**
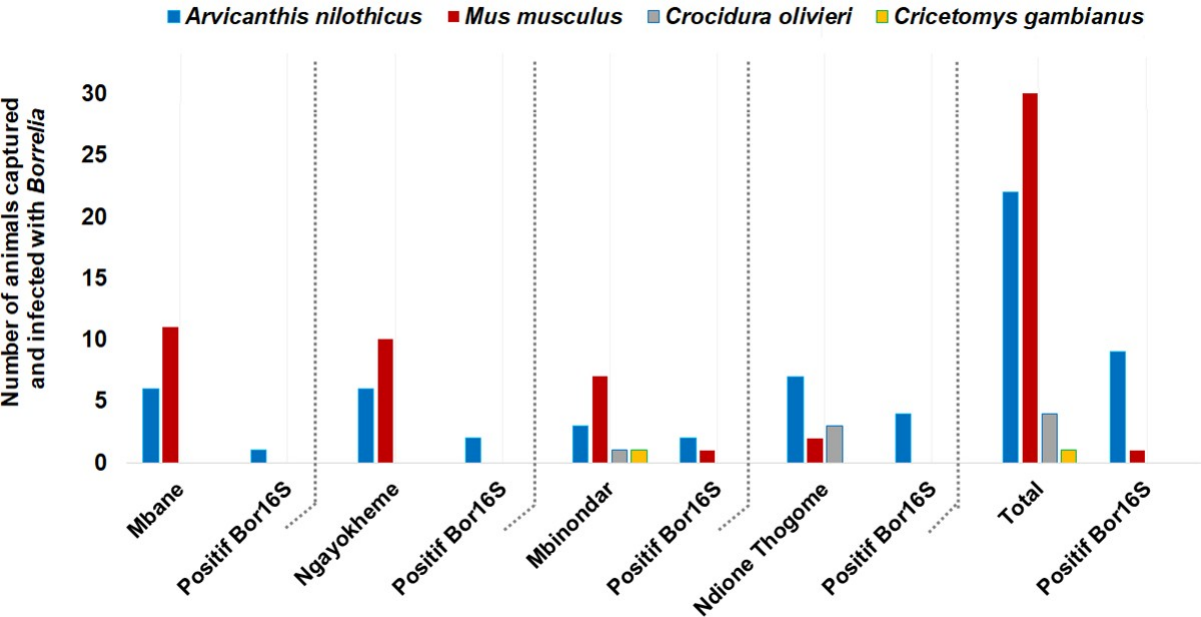
Numbers of animals captured and the prevalence of *Borrelia* infection in small mammals studied in the Niakhar district.

Overall, 67 small mammals (63 rodents and four insectivores) belonging to two different families and four different species, Muridae (*Arvicanthis niloticus*, *Mus musculus*, *and Cricetomys gambianus*) and Sorcidae (*Crocidura olivieri*) were caught in four villages (Fig 7). All animals were captured inside homes with a trapping yield of 7.1% (67/937).

Of the 67 small mammals captured, 57 were tested by qPCR amplification of brain DNA to detect *Borrelia* infection. The presence of *Borrelia* spp. infection was identified in 10 specimens (17.5%, 10/57) which belonged to two species (9/22 *A*. *niloticus* (40.9%) and 1/30 *M*. *musculus* (3.3%) collected from the villages studied (Table 3), including 1/17 *A*. *niloticus* in Mbane, 2/16 *A*. *niloticus* in Ngayokheme (12.5%), 3/12, comprising 2 *A*. *niloticus* and 1 *M*. *musculus*) in Mbinondar (25%), and 4/12 *A*. *niloticus)* in Ndione Thogome (33%).

**Table 3:** Prevalence of *Borrelia* infection in small mammals collected in the Niakhar district.

Species studied	Number of captures	Animals tested and infected with *Borrelia* (brain qPCR)
*Arvicanthis niloticus*	22	40.9% (9/22)
*Mus musculus*	40	3.3% (1/30)
*Crocidura olivieri*	4	0% (0/4)
*Cricetomys gambianus*	1	0% (0/1)
Total	67	17.5% (10/57)

## Discussion

The study findings indicate that the Niakhar district is a concerning focus of TBRF, with a high prevalence of infested rodent burrows, confirming that TBRF disease is a major cause of morbidity and is poorly diagnosed, as previous work performed in Senegal and other endemic areas of West and North Africa has concluded [1,3]. This lack of a diagnostic test to identify *Borrelia* infection in human patients using thick drop blood is due to the fact that there are practically no qualified technicians in the health posts and/or health centres who can act as a trained microscopic expert to examine *Borrelia* on the basis of a thick drop blood stained with Giemsa. Another reason is that there are no specialist technicians in the health posts and/or health centres who are able to detect *B*. *crocidurae* pathogens in febrile patients and rodent hosts using thick drop blood and/or rodent brains tissue by intraperitoneal inoculation into white mice, in addition to the fact that clinicians and physicians have little knowledge of TBRF. In this study, the proportion of *Ornithodoros* tick-infested burrows ranged from 13.2% to 66.6% depending on the village. A previous study reported 41%, 37% and 32% of positive burrows with a high proportion of infestation in three regions of Senegal located between 14°30’N and 15°30’N [7]. Recently, in the villages of Dielmo and Ndiop, 32% of investigated burrows (19/60) were found to be inhabited by *O*. *sonrai* ticks [5]. Although we were confident in our morphological identifications of the ticks to the species level, they have not been confirmed by molecular tools. However, the only species of soft tick that have been found to date in rodent burrows in Senegal are *O*. *sonrai* [1,3,8]. Other species of soft ticks such as *Ornithodoros capensis*, *O*. *maritimus* and *O*. *savignyi* have been found in bird nests and caves, but never in rodent burrows [35].

The prevalence of *Borrelia* infection in *O*. *sonrai* ticks was 12.7% (116/910), which was significantly higher for *O*. *sonrai* adult ticks (14.6%, 78/535) than nymphs (10.1%, 38/375). This could be explained by the fact that adult ticks feed several blood meals from different hosts who may harbour *Borrelia* and that the females lay eggs each time. In contrast, nymph feeds are performed by stasis (2–4 nymphal stases) with a moult occurring between each stasis varying from 8–21 days [4]. This result probably demonstrates the epidemiological importance of generations of *O*. *sonrai* infecting ticks from *O*. *sonrai* infected females that are involved in the transmission of TBRF through host feeding.

Quantitative real-time PCR is a powerful tool for detecting microorganisms from arthropods. It has been used to detect *Borrelia* species in many works. In our reference center, qPCR tests are considered positive when the cycle threshold (Ct) are lower than 36 [16,32]. Here, 26.7% DNA of ticks positive for the *16S_Bor* rRNA gene tested negative for the *B*. *crocidurae*-specific *GlpQ* gene. The qPCR Ct results for the *16S_Bor* gene and the specific *GlpQ* of *B*. *crocidurae* gene were similar, except for one sample. Similarly, in a study conducted in Senegal in 2020, 30.76% of rodents testing positive for *Borrelia* sp. using the *23S_Bor* gene for the screening were negative for the *GlpQ* gene [36]. The fact that ticks tested positive for the *16S_Bor* rRNA qPCR and negative with *B*. *crocidurae*-specific *GlpQ* qPCR, could be explained by the higher sensitivity of *16S_Bor* qPCR gene allowing to detect all *Borrelia* compared to *B*. *crocidurae* specific *GlpQ* qPCR gene or by the presence in the ticks of genetic variants of *B crocidurae*. This could also be explained by genetic variants of *B*. *crocidurae*, or indeed, the presence of other *Borrelia* that are not detected by the *GlpQ* gene assay.

However, this remains speculative and further study on the sensitivity and specificity of qPCR tests used to identify *Borrelia* spp. and the discriminatory power of *GlpQ* for speciation are needed. Testing the forward primer *16S_Bor* gene in silico, shows that it only matches bacteria of the *Borrelia* genus. However, interrogation of the reverse primer and the probe *in silico* show that they match in addition to bacteria of the *Borrelia* genus other species such as *Helicobacter* sp. and *Blautia* sp. Finally, the combination of the primers and the probe *16S_Bor* allows the detection of bacteria of the *Borrelia* genus but could also detect other bacterial species.This explains our strategy which consists in performing a qPCR using a gene targeting all bacteria of the genus in a first step, then in a second step a specific qPCR, such as in this work, or a standard PCR followed by sequencing to determine the bacterial species [16,17]. In this study, we used in the second step the *GlpQ* gene specific to *B*. *crocidurae* because we do not have a sequencing platform in Senegal. To our knowledge, only *B*. *crocidurae* and its many strains have been reported in *O*. *sonrai* ticks, Senegal and even in endemic West African countries [1,19,32,37].

In addition, for most work on *Borrelia* infection circulating in *O*. *sonrai* ticks and rodents in this part of West Africa, the TBRF endemic area only reports the presence of *B*. *crocidurae* associated with the *O*. *sonrai* tick vectors and hosted by rodents and insectivores [1,8,38,39,40]. However, the presence of other *Borrelia* spp. in other ticks can also not be excluded [41,42].

In Morocco, 41% of *Borrelia*-positive ticks were identified to carry *B*. *crocidurae* [35,40]. In previous studies conducted in Senegal, infection with *B*. *crocidurae* was reported in 26% of *Borrelia*-positive ticks [1] and in 21% of other *Borrelia*-positive ticks collected from 15 villages in the country and in adjacent areas of Mauritania and Mali along the 14^th^ parallel and the 12^th^ and 16^th^ meridians [3]. In Mali, 12.5% of *O*. *sonrai* ticks and 18% of other individuals were identified as carrying *B*. *crocidurae* [38], while in Tunisia, 15% of the small variety of *Ornithodoros erraticus* [25] and 6.5% of *O*. *sonrai* [1] were reported to carry *B*. *crocidurae*. Regarding the clinical study, we showed that TBRF is frequently diagnosed (12%) in the health structures in the Niakhar district. It has been demonstrated thatin the Sahelian and Sudanese regions, cases of TBRF occurred in all seasons, while in the Mediterranean areas there was a marked seasonality pattern in the distribution of TBRF cases, with most of them occurring between June and November in relation to higher temperatures [3,40]. There is no seasonality in the distribution of TBRF in Senegal as is the case in other endemic West African countries, due to the tropical climate which is marked by a dry and wet season with *O*. *sonrai* ticks being constantly active. Conversely, in the Mediterranean area located along the coast of the Mediterranean Sea, the climate is typically temperate with an autumn, winter and summer. During the winter, *O*. *sonrai* ticks are not active and hibernate, which explains why no cases of TBRF occurs during this period. In contrast, cases of TBRF were observed throughout the summer with a seasonal pattern in the distribution of human cases, which is not due to a depletion in alternative vertebrate hosts for *O*. *sonrai* ticks. In the winter, no tick biting activity can occur, which reflects the distribution of TBRF cases occurring during the autumn and summer is a result of seasonality. In *Borrelia*-infected patients, children between the ages of eight and 14 years old were the most infected by the disease (4.2% and 16%), as well as young adults aged between 15–19 and 20–29 (13.2% and 14.4%) respectively, as shown in previous studies [3,6,19]. This age group seems have much greater contact with the *O*. *sonrai* tick vector. *Borrelia* infection was largely found (17.5%) only in two species of rodents (*A*. *niloticus* and *M*. *musculus*), suggesting that these *Borrelia* hosting animals appear to play a major epidemiological role in the chain of transmission of the disease [3,6,7]. *Arvicanthis niloticus*, which most frequently hosts *Borrelia* infection in the wild is a species likely to be prolific around human dwellings [6,8,26]. In the Niakhar district, *A*. *niloticus* appears to be the main epidemiological host, causing the spread of *B*. *crocidurae* infection to *O*. *sonrai* ticks infecting humans. A recent study reporting *Borrelia* infection in small mammals from West Africa indicated that *A*. *niloticus* is the most frequently infected rodent in the wild followed by the other species of *Mastomys erythroleucus* [8] which were not captured during our study, but which are present in the district of Niakhar. In other TBRF endemic areas of Senegal (Dielmo, Dakar, Thiès and Richard-Toll), the species *A*. *niloticus* and *M*. *musculus* were found to be infected, with prevalence of *Borrelia* infection ranging respectively from 14% to 20% and 21% to 50% [3,6,9]. A rodent species which may be a main host for *B*. *crocidurae* hosts *O*. *sonrai* tick vectors, invertebrate reservoirs (due to their longevity and vertical transmission) involved in the spread and transmission of *Borrelia* infection from animal to animal and from animal to human [43].

Our results clearly indicate a sharp increase in the number of human cases detected between July and August in the Niakhar district, with a peak observed in August [44], corresponding to the period of high *O*. *sonrai* tick activity. In the village of Dielmo, the peak of TBRF occurrence was recorded in July during two years of monitoring [45]. It is surprising to note that during our field survey, the inhabitants did not know about TBRF, often considering that their state of illness was due to malaria, and reported never seeing the *O*. *sonrai* tick and didn’t know that this tick vector lived inside burrows in human dwellings. The considerable frequency of burrows (27.9%, 80/287) with infected *Ornithodoros* ticks in most of the villages that were studied suggests that TBRF is an endemic disease in the Niakhar district. The study showed a significant focus of TBRF infection in the Niakhar district and highlighted the fact that the *B*. *crocidurae* spirochete is likely to be the pathogenic agent causing febrile syndromes leading to consultations at these health posts and/or the health center. The wide spatial distribution of *O*. *sonrai* ticks in villages in the Niakhar district and the *Borrelia* infections detected both in *Ornithodoros* ticks and in small rodents and humans make the Niakhar district an endemic focus of TBRF. The lack of appropriate tools to diagnose TBRF makes health workers inefficient in terms of better dealing with all causes of fever relating to infective bites from *O*. *sonrai* tick vectors. The current high prevalence of TBRF in the Niakhar district suggests that in many other endemic areas of Senegal which have not yet been investigated (about 103 districts of the country are concerned by the presence of *Borrelia* infection), the disease is a major cause of morbidity which is usually undiagnosed in health posts and/or health centers which report unknown causes for spontaneous abortions in pregnant women [10–13], possible neurological complications, and jaundice in highly exposed young people [10–13]. TBRF is a neglected disease and should be taken into consideration more routinely by healthcare providers, public decision-makers and healthcare authorities to encourage appropriate antibiotic treatment in the event of unexplained fever which is frequently due to TBRF, and through the implementation of a national or West African programme of preventive control which would be referenced to the disease control strategy recently applied in two rural villages in the Fatick region [5]. This epidemiological surveillance will enable clinicians to pay more attention to common fevers of unknown origin such as TBRF and the reasons for consultation must be rigorously investigated for the benefit of the rural populations in endemic regions.

Lack of awareness of TBRF both among the populations affected and the wider medical community including the medical training of students, health workers and physicians is a major but avoidable problem which deserves greater consideration from public authorities and decision-makers. Challenges remain to be overcome. This is a key priority area when it comes to reducing the clinical burden of this treatable infection occurring within endemic areas of rural communities in Senegal and other West African countries. Our findings reveal the major involvement of *O*. *sonrai* ticks and mammalian reservoir hosts in the transmission of TBRF, a neglected public health problem.

We believe that the results of our study should strongly encourage health authorities to include doxycycline in decision trees for the treatment of fevers of unknown origin.

## Supporting information

S1 Table Detailed results of *Ornithodoros sonrai* ticks sampled in August 2016 and infected ticks surveyed; in the Niakhar district, Senegal. (DOCX)
