# Supplementary material for: Tick-borne relapsing fever Borreliosis, a major public health problem overlooked in Senegal
Source: PLoS Negl Trop Dis. 2021 Apr 22;15(4):e0009184. doi: 10.1371/journal.pntd.0009184 (PMC8096072; doi:10.1371/journal.pntd.0009184)
Supplement: S1 Table — (DOCX) [file pntd.0009184.s001.docx]

**Supplementary Table**. Detailed results of *Ornithodoros sonrai* ticks sampled in August 2016 and infected ticks surveyed; in the Niakhar district, Senegal

| **Geographical coordinates** | **Villages included** | **Number of burrows examined** | **Number of infested burrows** | **Infestation rate** | ***Borrelia* spp. / *B. crocidurae* infected ticks** |
| --- | --- | --- | --- | --- | --- |
| 14°21'N-16°24'W | Mbine Diene | 15 | 2 | 13.2% | 0/12 (0%) |
| 14°22'N-16°25'W | Mbane | 30 | 10 | 33.3% | 3/61 (4.9%) |
| 14°22'N-16°23'W | Nguess | 22 | 7 | 31.8% | 5/16 (31.2%) |
| 14°22'N-16°22'W | Keur Ama Sarr | 10 | 3 | 30% | 3/7 (42.8%) |
| 14°24'N-16°24'W | Sagne | 30 | 14 | 46.6% | 1/84 (1.2%) |
| 14°24'N-16°26'W | Koneme | 25 | 4 | 16% | 1/18 (5.5%) |
| 14°24'N-16°21'W | Mbine Ndoup | 15 | 10 | 66.6% | 4/25 (16%) |
| 14°24'N-16°22'W | Keur Mang Mbaye | 15 | 8 | 53.3% | 6/35 (17.1%) |
| 14°25'N 16°24'W | Mbafaye | 10 | 8 | 80% | 7/22 (31.8%) |
| 14°25'N 16°22'W | Bibane | 10 | 3 | 30% | 0/3 (0%) |
| 14°26'N 16°26'W | Mbine Waly Sikat | 10 | 1 | 10% | 0/1 (0%) |
| 14°26'N 16°21'W | Yayeka | 10 | 8 | 80% | 4/16 (25%) |
| 14°27'N 16°24'W | Ngolgane | 30 | 4 | 13.3% | 2/12 (16.6%) |
| 14°27'N 16°26'W | Kobosikil | 30 | 10 | 33.3% | 1/32 (3.1%) |
| 14°27'N 16°21'W | Ndoss Diaraf | 30 | 10 | 33.3% | 9/39 (23%) |
| 14°27'N-16°23'W | Langheme | 30 | 16 | 53.3% | 3/33 (9%) |
| 14°28'N-16°26'W | Mbinondar | 11 | 9 | 81.8% | 7/31 (22.6%) |
| 14°28'N-16°24'W | Sassar | 12 | 4 | 33.3% | 0/8 (0%) |
| 14°28'N-16°22'W | Toka Sone | 15 | 1 | 6.6% | 0/1 (0%) |
| 14°28'N-16°19'W | Sambang | 10 | 4 | 40% | 3/20 (15%) |
| 14°29'N-16°24'W | Ndianeme | 15 | 6 | 40% | 1/22 (4.5%) |
| 14°29'N-16°26'W | Diokoul | 30 | 6 | 20% | 0/22 (0%) |
| 14°30'N-16°21'W | Lahar | 30 | 9 | 30% | 4/13 (30.7%) |
| 14°30'N-16°24'W | Sass Ndiafadji | 30 | 13 | 43.3% | 5/31 (16.1%) |
| 14°30'N-16°26'W | Ngane Fissel | 25 | 7 | 28% | 2/18 (11.1%) |
| 14°30'N-16°27'W | Lambaneme | 30 | 14 | 46.6% | 7/38 (18.4%) |
| 14°31'N-16°18'W | Tella Yargouye | 10 | 4 | 40% | 3/18 (16.6%) |
| 14°31'N-16°26'W | Ngayokheme | 10 | 6 | 60% | 0/12 (0%) |
| 14°32'N-16°29'W | Toucar | 10 | 7 | 70% | 0/20 (0%) |
| 14°32'N-16°21'W | Mbadatte | 15 | 5 | 33.3% | 5/20 (25%) |
| 14°32'N-16°24'W | Wakhaldiam | 30 | 17 | 56.6% | 9/41 (21.9%) |
| 14°32'N-16°28'W | Poudaye | 30 | 9 | 30% | 0/23 (0%) |
| 14°33'N-16°18'W | Beredji | 15 | 7 | 46.6% | 2/26 (7.7%) |
| 14°33'N-16°21'W | Ndiambour | 15 | 4 | 26.6% | 0/8 (0%) |
| 14°34'N-16°21'W | Ndione Thogome | 10 | 7 | 70% | 4/22 (18.2%) |
| 14°34'N-16°24'W | Diadiack | 10 | 2 | 20% | 0/3 (0%) |
| 14°34'N-16°26'W | Ngalagne Kop | 15 | 5 | 33.3% | 3/21 (14.3%) |
| 14°34'N-16°29'W | Ngangarlame | 13 | 0 | 0% | 0/0 (0%) |
| 14°35'N-16°30'W | Bari Sine | 25 | 8 | 32% | 4/18 (22.2%) |
| 14°35'N-16°27'W | Datel | 30 | 14 | 46.6% | 7/39 (17.9%) |
| 14°35'N-16°24'W | Samba Toude | 30 | 5 | 16.6% | 0/8 (0%) |
|  | Total | 788 | 287 | 36.4% | 116/910 (12.7%) |
